# The role of recombination landscape in species hybridisation and speciation

**DOI:** 10.3389/fpls.2023.1223148

**Published:** 2023-07-06

**Authors:** Edgar L. Y. Wong, Dmitry A. Filatov

**Affiliations:** ^1^ Department of Biology, University of Oxford, Oxford, United Kingdom; ^2^ Senckenberg Biodiversity and Climate Research Centre, Frankfurt am Main, Germany

**Keywords:** introgression, recombination, evolution, hybridisation, speciation, gene flow

## Abstract

It is now well recognised that closely related species can hybridize and exchange genetic material, which may promote or oppose adaptation and speciation. In some cases, interspecific hybridisation is very common, making it surprising that species identity is preserved despite active gene exchange. The genomes of most eukaryotic species are highly heterogeneous with regard to gene density, abundance of repetitive DNA, chromatin compactisation etc, which can make certain genomic regions more prone or more resistant to introgression of genetic material from other species. Heterogeneity in local recombination rate underpins many of the observed patterns across the genome (e.g. actively recombining regions are typically gene rich and depleted for repetitive DNA) and it can strongly affect the permeability of genomic regions to interspecific introgression. The larger the region lacking recombination, the higher the chance for the presence of species incompatibility gene(s) in that region, making the entire non- or rarely recombining block impermeable to interspecific introgression. Large plant genomes tend to have highly heterogeneous recombination landscape, with recombination frequently occurring at the ends of the chromosomes and central regions lacking recombination. In this paper we review the relationship between recombination and introgression in plants and argue that large rarely recombining regions likely play a major role in preserving species identity in actively hybridising plant species.

## 1 The role of recombination in reproductive isolation

Understanding how new species form and how reproductive isolation evolves are important long-standing topics in evolutionary biology. There are numerous types of speciation that comes with different types of reproductive isolation – pre- or post-zygotic isolation, phenotypic, genomic or geographical isolation, asexual or sexual isolation, and so on. Recombination has long been hypothesised to play a significant role in determining the rate of speciation, hybridisation and adaptation (Ortíz-Barrientos et al., 2002; Butlin, 2005, Sousa et al., 2013). Better understanding of the distribution of recombination along the genome provides insights into how reproductive isolation evolves and how selection acts on introgression (Martin and Jiggins, 2017). It may help to improve crop breeding, in which low-recombining regions have been a major obstacle in creating more productive crop breeds (Bai and Lindhout, 2007; Soyk et al., 2019; Fuentes et al., 2022a).

### 1.1 Recombination versus divergence

The concept of suppression of recombination as the basis of divergence maintenance is not new, with studies examining both collinear genomes (Butlin, 2005; Feder et al., 2012a, b), and genomes with rearrangements in form of structural variants and chromosomal inversions (e.g. Roesti et al., 2014; Twyford and Friedman, 2015; Moyers et al., 2018; Todesco et al., 2020; Fuentes et al., 2022b). Felsenstein (1981) had proposed that in the speciation with gene flow model, the role of suppressed recombination is to secure linkage disequilibrium between locally adaptive alleles and those for non-random mating. The model also anticipates that genomic regions with lower rates of recombination would harbour targets for reproductive isolation (Nachman and Payseur, 2012). Other studies have proposed that in hybridising species or populations, there is a tendency for reproductive isolation to concentrate in regions with low recombination (Butlin, 2005; Payseur and Rieseberg, 2016).

In general, recombination breaks up species- or population-specific allelic combinations, which reduces genetic differentiation. However, there are many examples of actively hybridising species or introgressed populations that maintain their genetic identities without them merging into a single lineage (e.g., *Helianthus*: Owens et al., 2016; Owens et al., 2021; *Senecio*: Wong et al., 2020; Wong et al., 2022; Wong et al., 2023; *Silene*: Muir et al., 2012; Hu and Filatov, 2016; Filatov, 2018; Karrenberg et al., 2019). In these species, certain genomic regions help to maintain species identity. These include pericentromeric regions where crossover rates are inherently lower, regions which harbour inversions (that reduce recombination in heterozygotes), and Dobzhansky–Muller incompatible (DMI) alleles. For DMI alleles, strong negative epistasis and thus selection for allelic modifiers could favour lower recombination (Lenormand and Otto, 2000). These modifiers would then spread in their respective populations and contribute positively to genetic divergence (Ortiz-Barrientos et al., 2016). The resulting blocks of co-adapted alleles could be seen as a form of reinforcement, since they reduce the survival of offspring with heterozygous alleles and modifiers, especially during secondary contact and early stages of speciation (Ortiz-Barrientos et al., 2016). Low-recombining regions also often harbour clusters of barrier loci between diverging populations as existing barrier loci could shield newly established ones in close linkage (Rafajlović et al., 2016) and these clusters could potentially promote the evolution of low-recombining regions (Yeaman, 2013).

**Figure 1:**
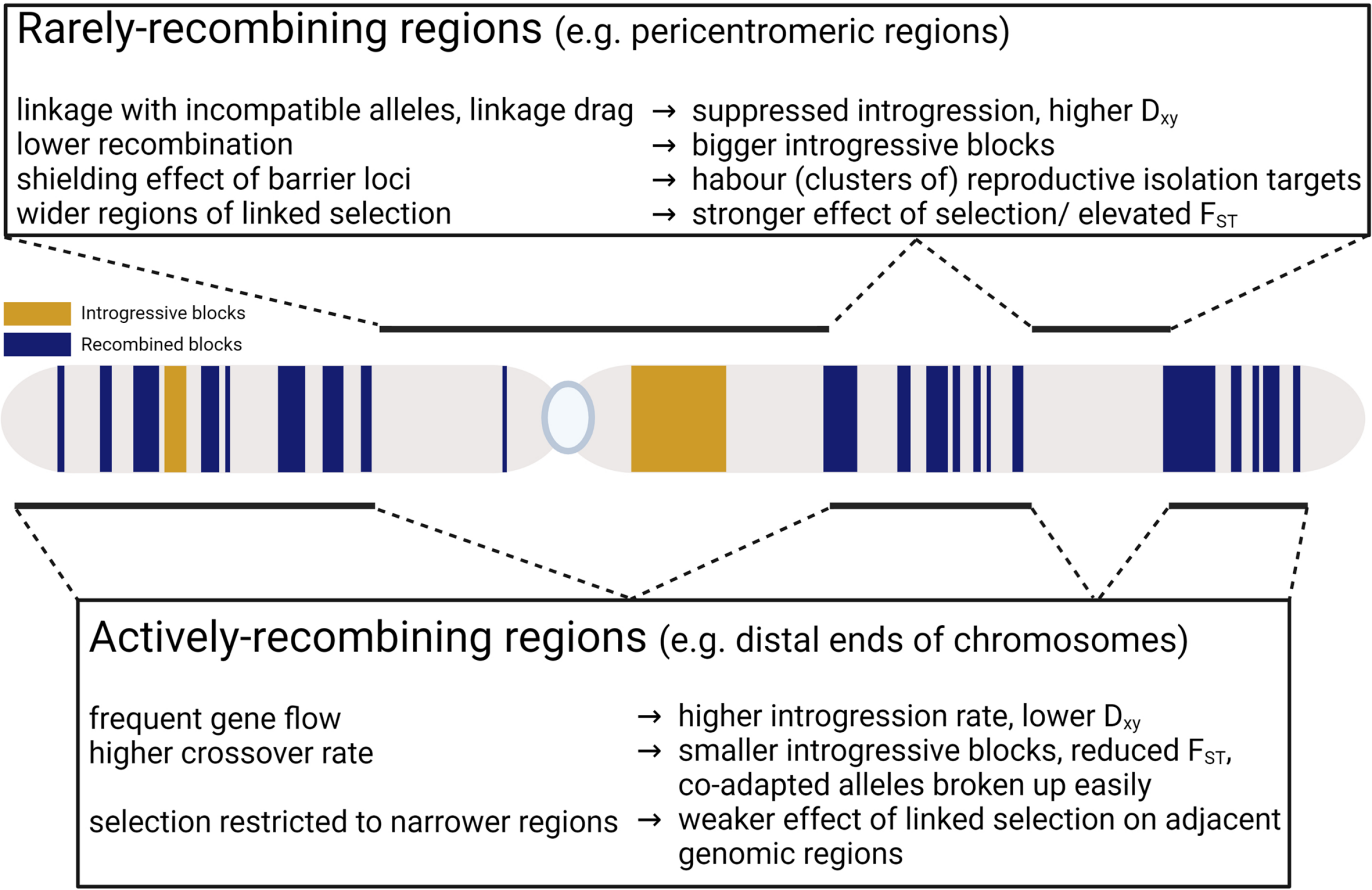
Contrasting processes involved in rarely- and actively-recombining regions.

Population differentiation, measured as F_ST_ for example, has been demonstrated to negatively correlate with recombination rate (e.g. Baines et al., 2004; Takahashi et al., 2004; Keinan and Reich, 2010). Positive and negative selection could both create this pattern (Nachman and Payseur, 2012). F_ST_ shows the proportion of total polymorphism that is due to divergence between the two species (Nei, 1987). Reducing intra-specific variation or increasing inter-specific divergence both increase F_ST_ (**Figure 1**). Positive selection for different alleles in the two species increases divergence and reduces intra-specific variation at the selected site and the adjacent linked sites. Negative selection can elevate F_ST_ by reducing intra-specific genetic diversity due to ‘background selection’ – elimination of deleterious alleles leading to reduction of the effective population size at alleles linked to the selected ones (Charlesworth, 1998), but the effects of positive selection (selective sweeps) are expected to be more rapid than that of negative selection (Keinan and Reich, 2010). Fluctuating selection may also reduce diversity (Gillespie, 1994; Barton, 1995), but it can also lead to long-term maintenance of polymorphisms by balancing selection (Charlesworth, 2006), which increases intraspecific diversity and thus reduces F_ST_. The effects of selection on population differentiation are expected to be stronger in regions of low recombination as recombination breaks down non-random associations, limiting the effect of hitchhiking or background selection (linked selection) to a narrower genomic region. With lower recombination the regions affected by linked selection are wider and selection (or processes resembling selection, such as meiotic drive) would reduce intra-specific nucleotide diversity in a wider genomic region, causing the negative correlation between recombination and F_ST_ (Nachman and Payseur, 2012; Cruickshank and Hahn, 2014).

For other population divergence indices, such as D_xy_, their relationship with recombination is more nuanced (**Figure 1**). D_xy_ is the average sequence difference between individuals in two populations or species (Nei, 1987). It is mostly unaffected by neutral alleles that are in linkage disequilibrium with deleterious ones if gene flow is low in low-recombining regions (e.g. Charlesworth et al., 1997; Noor and Bennett, 2009; Cruickshank and Hahn, 2014; Zeng and Corcoran, 2015), unlike F_ST_ (and other relative differentiation indices) which would be elevated as a result. High recombination rate could result in low D_xy_ due to frequent gene flow; however, low D_xy_ could also be observed in regions of low recombination if linked selection operated in ancestral population causing shorter coalescent time for alleles in the diverging populations (Nachman and Payseur, 2012).

### 1.2 Recombination versus introgression

Theory and many empirical studies support a positive correlation between recombination rate and introgression (e.g. Butlin, 2005; Noor and Bennett, 2009; Nachman and Payseur, 2012; Brawand et al., 2014; Samuk et al., 2017; Schumer et al., 2018; Martin et al., 2019). Frequently-recombining regions, such as chromosome ends away from the centromere, are more penetrable for interspecific gene flow, as higher recombination rate would decouple compatible introgressing alleles from incompatible ones (Barton and Bengtsson, 1986; Gante et al., 2016; Martin and Jiggins, 2017) and allow common alleles to segregate in hybridising species (Felsenstein, 1981; Feder and Nosil, 2010; Nachman and Payseur, 2012; Ravinet et al., 2017; Wolf and Ellegren, 2017). In rarely-recombining regions, interspecific incompatibilities are not the only barrier to introgression. Selection against introgression in these regions could also stem from linkage drag – the segregation of weakly deleterious mutations in populations that have smaller population sizes (Harris and Nielsen, 2016; Juric et al., 2016). Introgression is likely suppressed around loci responsible for reproductive isolation (Nosil, 2012; Ravinet et al., 2017; Elmer, 2019), as well as islands of differentiation (Feder and Nosil, 2010; Flaxman et al., 2012; Flaxman et al., 2013). Introgression levels could also be impeded due to nucleo-cytoplasmic interactions. Nucleo-cytoplasmic incompatibilities have been reported in many species, in which one introgression direction results in higher fitness in offspring than the other (e.g., Leinonen et al., 2011; Brennan et al., 2014; Senerchia et al., 2015; Senerchia et al., 2016; Zuellig and Sweigart, 2018; Wong et al., 2023). As cytoplasmic genomes are typically non-recombining, they present significant barriers to introgression.

Although research on the interplay between recombination and introgression centres around animals, such as house mice (Janoušek et al., 2015), humans (Harris and Nielsen, 2016; Juric et al., 2016; Schumer et al., 2018), swordtail fish (Schumer et al., 2018) and stickleback (Ravinet et al., 2018, Ravinet et al., 2021), research on the relationship between recombination and introgression in plants is not entirely lacking. In barley, variation in recombination rate along the genome had stronger effects on patterns of introgression than genome-wide differences in recombination among populations (Dreissig et al., 2020). In wheat, it was shown that rarely-recombining regions possess more potentially deleterious single nucleotide polymorphisms (SNPs) than frequently-recombining ones due to Hill-Robertson effects (Hill and Robertson, 1966; Jordan et al., 2018). These findings seem to agree that rates of recombination and introgression are correlated and suggest that selection against introgression in rarely-recombining regions is strong enough to resist genetic merger between hybridising species or populations despite frequent genetic exchange that leads to reduced divergence in other regions. It is worth noting that recombination rate in the same genomic region may not be constant across the species’ range. Dreissig et al. (2019) examined wild barley populations grown in different environmental conditions and revealed that recombination rate has a positive, linear relationship with precipitation; whereas mean annual temperature, isothermality and solar radiation all shared a non-linear relationship with recombination rate.

## 2 Evidence for the role of pericentromeric regions in reproductive isolation among introgressing species

Analysing species differentiation across the genomes in five pairs of recently diverged species, islands of divergence were identified in pericentromeric or peritelomeric regions, which had lower recombination rates (Cruickshank and Hahn, 2014) and occasionally resulted in lengthy regions of reduced diversity (Cutter and Payseur, 2013). In hybridising butterflies, genomic regions with low recombination rates (≤5 cM/Mb) were found to have reduced admixture (Martin et al., 2019). On top of that, highly-differentiated speciation islands located in pericentromeric regions on two chromosomes were found to be the most probable cause for reproductive isolation in *Anopheles gambiae* ecotypes (Costantini et al., 2009); while another study concluded that the X chromosome’s pericentromeric regions is the only region that differentiates M and S forms of *A. gambiae* (Nwakanma et al., 2013) and that selection is likely responsible for limiting gene flow in this region to maintain divergence (Caputo et al., 2011; Marsden et al., 2011; Weetman et al., 2012; Nwakanma et al., 2013). Many plant studies have also demonstrated that recombination rate is lower in pericentromeric regions, including in common beans (Bhakta et al., 2015), wheat (Jordan et al., 2018), barley (Dreissig et al., 2019; Dreissig et al., 2020), rice (Fayos et al., 2022) and white campion (Filatov, 2023). Others have shown that crossover rate (which is directly correlated with recombination rate) is lower in these regions in tomatoes (Demirci et al., 2017; Fuentes et al., 2020, Fuentes et al., 2022b) and wheat (Jordan et al., 2018). Nyine et al. (2020) also showed that introgression is less frequent in low-recombining regions in winter wheat and its relatives.

However, some studies found a negative relationship between introgression level and recombination rate (e.g. Dreissig et al., 2020; Duranton and Pool, 2022). Duranton and Pool (2022) suspected that the negative relationship they observed stemmed from positive selection; while others have demonstrated that introgression from a smaller to larger population would produce the same relationship (as seen in introgression from Neanderthals to modern humans: Harris and Nielsen, 2016; Juric et al., 2016; Kim et al., 2018). Some studies also found that pericentromeric regions do not habour elevated divergence, leading the authors to suggest that divergence is maintained according to the arrangement of functional elements rather than recombination rate (e.g. Wersebe et al., 2023). The size of introgressed regions tends to be bigger in pericentromeric compared to distal chromosome regions (Dreissig et al., 2020), as expected due to more extensive linkage disequilibrium in rarely recombining regions. Introgression in barley is rapidly driven to fixed homozygosity, as expected for a strictly self-fertilising species (Dreissig et al., 2020). Crossovers, recombination and introgression were also found to be nearly absent in the centromeric and pericentromeric regions in *Mimulus cardinalis* (Nelson et al., 2021) and tomatoes (Demirci et al., 2017; Fuentes et al., 2020) (with the exception of scattered recombination hotspots in these regions: Fuentes et al., 2022b).

## 3 Challenges in identifying underlying cause of divergence

As multiple factors could create the negative correlation between population differentiation and recombination rate, one major challenge in speciation studies is to distinguish which of the factor(s) caused the observed patterns in various systems. In particular, did restricted gene flow or selection cause high F_ST_ at the centromeric regions (Nachman and Payseur, 2012)? For example, Takahashi et al. (2004) suggested that either background selection or selective sweeps could be the cause of the negative correlation between population differentiation and recombination rate they observed in a structured population of mice. Another challenge is to narrow down target regions underlying reproductive isolation to smaller regions or even individual causative functional genes and nucleotide polymorphisms (Ravinet et al., 2017). Distinguishing between barrier and non-barrier loci is not trivial either, particularly so in the rarely recombining regions. This is because strong linkage disequilibrium between non-barrier and barrier loci make it difficult to identify which of the loci in a non-introgressing region is preventing population-specific alleles to spread to other populations (Ravinet et al., 2017). Currently, most studies use a window-based approach which assumes constant recombination rate within each window (Payseur and Rieseberg, 2016). This does not reflect the real pattern as recombination rate fluctuates on multiple scale (e.g. Comeron et al., 2012; Liu et al., 2014). Variation of recombination rate across the species range (e.g. Dreissig et al., 2019) is another problem that has to be taken into account in the analyses. Clearly, examining the recombination landscape and its effect on interspecific gene flow at a finer scale remains a challenge for future research.

## 4 Conclusion

There is substantial evidence that low-recombining genomic regions show higher species differentiation compared to actively recombining regions, which suggests that the former plays a significant role in speciation and contributes to restricting gene flow between hybridising taxa. However, the role of rarely recombining regions in speciation is not fully understood. Rarely recombining regions may play a significant role in maintenance of species identity in actively hybridising species (e.g. Ortiz-Barrientos et al., 2016; Payseur and Rieseberg, 2016). Alternatively, the rarely recombining regions may stand out as the hotspots of species differentiation only because stronger linked selection in such regions inflates the measures of differentiation, such as F_ST_ (Cruickshank and Hahn, 2014). More evidence of enrichment of rarely recombining regions for genes responsible for species-specific traits is needed to confirm their specific role in the maintenance of species identity in face of interspecific gene flow. Furthermore, instead of being the cause, reduced recombination could be a consequence of diversifying selection during speciation with gene flow (Ortiz-Barrientos et al., 2016) or, in other words, selection for alleles responsible for local adaptation (e.g. Kirkpatrick and Barton, 2006; Yeaman, 2013). Future research is needed to clarify these relationships between recombination rate and divergence. It will also be important to extend the analyses to a wider range of species to diversify study systems, especially in the plant kingdom, where many hybridising species are available for detailed analysis of interspecific introgression in the context of recombination landscape across the genome.

## Author contributions

EW and DF came up with the concept of this mini-review. EW wrote the initial draft. Both authors contributed to editing and approved the submitted version.
